# Influence of navigation system updates on total knee arthroplasty

**DOI:** 10.1186/2052-1847-5-10

**Published:** 2013-05-02

**Authors:** Hiroshi Inui, Shuji Taketomi, Kensuke Nakamura, Seira Takei, Hideki Takeda, Sakae Tanaka, Takumi Nakagawa

**Affiliations:** 1 Department of Orthopaedic Surgery, Faculty of Medicine, The University of Tokyo, Tokyo, Japan

**Keywords:** Image-free navigation, Navigation system update, Total knee arthroplasty, Component positioning, Operating time, Clinical outcome

## Abstract

**Background:**

The purpose of this study was to evaluate the influence of image-free computer-assisted navigation system update on outcome in total knee arthroplasty.

**Methods:**

Thirty-three knees were replaced using the Stryker 3.1 image-free navigation system and 49 knees were replaced using the Stryker 4.0 system. One surgeon took part in all procedures as chief surgeon or first assistant. All patients received the Stryker Scopio NRG CR total knee prosthesis. We compared the accuracy of component positioning measured using radiographs and CT scans, operating time and clinical outcome 1 year after surgery.

**Results:**

The mean hip-knee-ankle, frontal femoral and tibial component angle were 179.8° (ideally implanted 85%), 89.8° (88%), 90.4° (88%) respectively for the 3.1 group and 179.5° (96%), 90.6° (92%), 90.2° (94%) for the 4.0 group. The mean sagittal tibial component angle was 85.5° (82%) for the 3.1 group and 85.6° (92%) for the 4.0 group. The mean rotational femoral and tibial component angle were −0.5° (81%), −0.7° (73%) for the 3.1 group and 0.0° (84%), 0.4° (72%) for the 4.0 group. There were no statistically significant findings with regard to component positioning.

Operating time was significantly longer in the 3.1 group (3.1 group: 137 min, 4.1group: 125 min, P < 0.01).

No significant difference was detected in postoperative clinical outcome.

**Conclusion:**

The navigation system update from Stryker 3.1 to Stryker 4.0 reduced operating time by 12 min. However, there were no statistically significant findings with regard to component positioning and clinical outcome.

## Background

Total knee arthroplasty (TKA) has become one of the most successful surgical procedures in orthopedic surgery [1,2]. The success of this procedure depends on many factors, including surgical techniques and the design and material of the components. With regard to surgical techniques, implant positioning and soft tissue balancing are very important. Malpositioning of any component can lead to an increased risk of loosening, instability, and pain [3,4]. Restoration of the tibiofemoral angle to within 3° of neutral during TKA is thought to be associated with better outcome [4-7]. The accurate rotational alignment of femoral and tibial components is also considered important [3,8,9].

Computer-assisted navigation systems are designed to increase the accuracy of implantation, and have become much more accepted and prevalent in recent years. Several studies, including a meta-analysis study have demonstrated superior alignment of the components in the coronal plane in navigated compared with conventional implanted TKA, with fewer outliers outside a range of 3° varus or valgus [7,10,11]. Some studies have noted an improvement in the accuracy of rotational alignment using navigation systems [12,13]. However, it is still not clear whether navigation can improve rotational alignment consistently [14,15].

Recently, several software and adapted instrument advancements have been made to further improve the accuracy of total knee component positioning. One recent study demonstrated that advancements in navigation software improved the accuracy of overall mechanical alignment and several individual component positioning variables; however rotational alignment was not evaluated in that study [16]. Although consistent and accurate rotational positioning is desirable, it remains to be elucidated whether currently available navigation software improves rotational accuracy of placement of components.

Version 2.0 of the Stryker image-free navigation system was first implemented in our institute in January 2007. It was updated to version 3.1 in July 2007 and version 4.0 in January 2009. Molli et al. [16] demonstrated several advancements of the 3.1 system over the 2.0 system, which led to more accurate implant positioning. Several advancements of the 4.0 system have also been noted. Therefore, we hypothesized that this recent navigation system update would improve alignment in TKA and reduce operating time. We evaluated several outcome measurements including overall alignment, individual component positioning, knee score, range of motion, and operating time.

## Methods

This study was approved by the institutional review boards of the University of Tokyo (No.2674). All patients provided written informed consent.

Of a total of 109 consecutive primary TKA procedures performed in 93 patients between December 2007 and March 2009, 46 consecutive knees in 33 patients were replaced using the Stryker 3.1 image-free computer navigation system (Stryker Orthopedics, Mahwah, NJ, USA) between December 2007 and December 2008. Sixty-three consecutive knees in 60 patients were replaced using the updated Stryker 4.0 image-free navigation system between January 2008 and March 2009. Thirty-three knees in 30 patients of the Stryker 3.1 group and 49 knees in 48 patients of the Stryker 4.0 group met the inclusion criteria: no major previous orthopedic surgeries (i.e.,, arthroplasty, open reduction-internal fixation procedures, osteotomy), satisfactory full-length standing anteroposterior (AP) and lateral radiographs after operation, complete data entry, and adequate follow up of minimum 1 year. Preoperative variables were recorded including age, sex, body mass index, preoperative diagnosis, mechanical axis, and range of motion. Pre-operative scores were obtained using the Knee Society Score (KSS) [17]. There was no statistically significant difference between the groups in terms of demographic features (Table 1).

**Table 1:** Pre-operative demographic data

	**Version 3.1 group**	**Version 4.0 group**	**P-value**
Number of patients	33	49	
Sex (female/male)	30/3	43/6	
Diagnosis (OA/others)	31/2	47/2	
Age (years)	75.0 ± 5.0	75.5 ± 5.1	n.s.
Pre-operative KSS	37.9 ± 10.5	36.4 ± 10.1	n.s.
Maximum extension (°)	−9.8 ± 7.2	−11.8 ± 7.1	n.s.
Maximum flexion (°)	120.1 ± 16.4	119.2 ± 15.7	n.s.
Body mass index (kg/m^2^)	26.2 ± 3.7	26.1 ± 4.1	n.s.
Pre-operative FTA (°)	185.8 ± 6.9	186.1 ± 9.6	n.s.

### Operating procedures

The Stryker Navigation System was used for computer-assisted implantation. The system was image-free and used infrared cameras and light-emitting diodes.

Surgery was performed under a tourniquet. A midvastus approach was used for the varus and neutral knee and a medial parapatellar approach was used for the valgus knee. Bicortical tracker pins were placed into the femoral shaft at the proximal end of the skin incision and the tibial shaft at the distal end of the skin incision. Anatomical landmarks were registered either by the pointer, the validation of which is successful if the deviation between the pointer tip position and the calibration data is 2 mm, or by the calculations of the navigation system to proceed with bone resections and implant positioning. Landmarks comprised the center of the femoral head, the distal femur, the proximal tibia and the ankle, the Whiteside line, the epicondylar axis (lateral epicondyle, medial sulcus), anterior surface of the distal femur cortex, the condylar surfaces of the femur and tibia, and the tibia AP axis.

The center of the femoral head was determined by rotating the femur by rotational calculations. The center of the ankle was represented by a 44%–56% medial to lateral ratio along the transmalleolar axis.

Femoral alignment was aimed at a placement of 90° to the mechanical axis in the frontal plane. In the sagittal plane, anterior notching was avoided by changing the flexion angle and implant size. The femoral rotation axis was defined as the average rotation axis of the transepicondylar axis and the axis perpendicular to the Whiteside line.

For the tibia, alignment was aimed at 90° to the mechanical axis in the frontal plane and 5° of posterior slope in the sagittal plane. The AP axis was aimed along the line from the medial border of the tibia tubercle to the middle of the posterior cruciate ligament [18]. To avoid registration error, we drew the line in blue beforehand.

During femoral and tibial resection, the femur was prepared first. Care was taken to balance the flexion and extension gaps and release any flexion contracture. The patella was everted and resurfaced in all patients. All patients underwent the same postoperative rehabilitation protocol.

**Figure 1:**
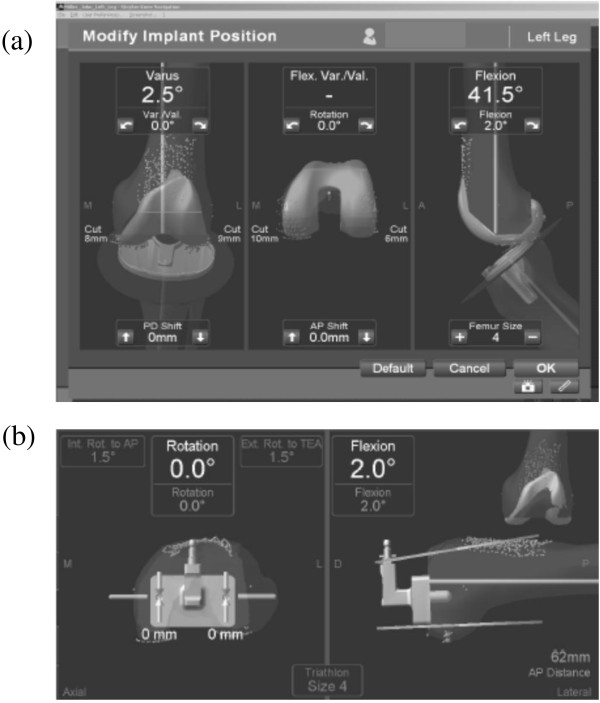
**Automatic templating system. a**. Automatic implant sizing and positioning system. **b**. Navigation template drilling system, which maintains the registered femoral rotation angle until setting of the 4-in-1 cutting guide.

There were several advancements of the 4.0 system over the 3.1 system. The number of sensors in the camera was increased from two to three, which facilitated faster and more accurate registration [19]. The automatic implant sizing and positioning system provided accurate implant sizing and aided in avoiding anterior femoral notching (Figure 1a). The navigation template drilling system maintained the registered femoral rotation angle until setting of the 4-in-1 cutting guide and eliminated unnecessary procedures (Figure 1b).

One surgeon (TN) took part in all procedures as chief surgeon or first assistant. All implants used in both groups were Stryker Scopio NRG CR total knee implants. Identical cutting blocks and instrumentation devices were used for all procedures.

### Evaluation of post-operative alignment

**Figure 2:**
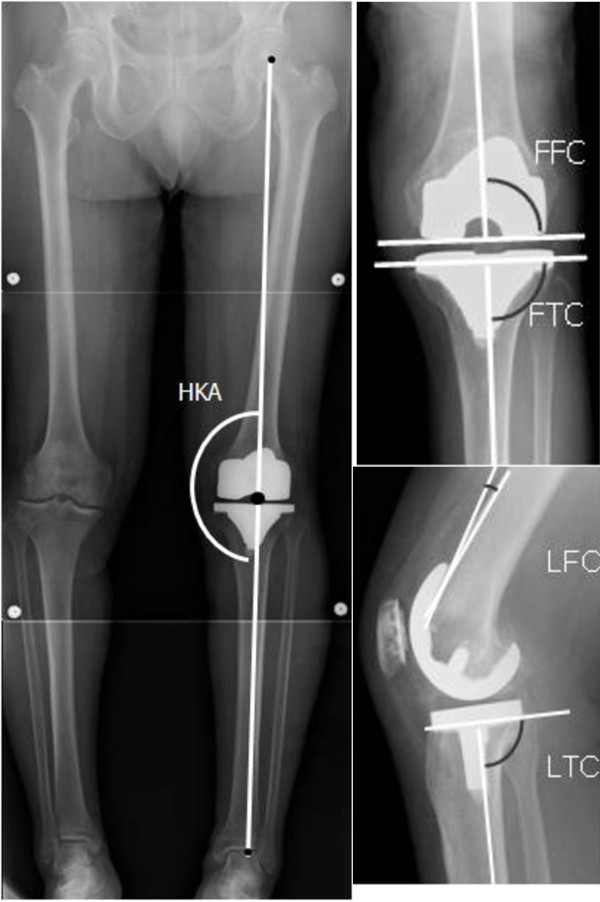
Evaluation of coronal and sagittal alignment by full-length standing and lateral radiography.

Alignment in the frontal plane was measured using full-length standing radiograph performed 3–6 months after operation [20]. The mechanical axis of the leg was defined as the hip–knee–ankle (HKA) angle, which is the angle between the line connecting the center of the hip with that of the knee (the mechanical axis of the femur) and the line connecting the center of the knee with that of the ankle joint (the mechanical axis of the tibia). Frontal alignment of femoral and tibial components (FFC: frontal femoral component angle, FTC: frontal tibial component angle) were also measured by full-length radiograph. The femoral component sagittal alignment and tibial slope (LFC: lateral femoral component angle, LTC: lateral tibial component angle) were measured by lateral radiograph (Figure 2). The LFC angle was measured between the anterior cortex of the distal femur and the shield of the femoral component. The ideal mechanical axis was defined as falling within 3° of 180°, the ideal frontal femoral and tibial component angles were within 2° of 90°, and the ideal sagittal tibial angle was within 2° of 85° [11,12,16]. The sagittal femoral component angle was determined separately in each individual through minute change in flexion angle and implant size for avoiding anterior notching.

**Figure 3:**
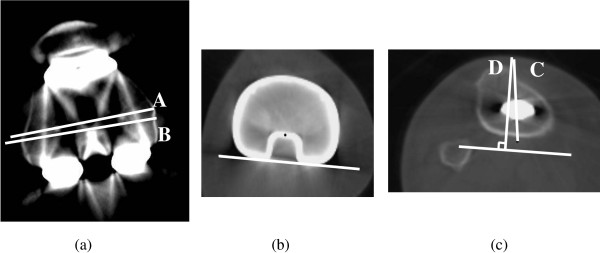
**Measurement of rotational alignment. a**. RFC angle is defined as the angle between **A** and **B**. **A**: Line through the center of both fixation pegs. **B**: Surgical epicondylar line. **b**. White line: along the posterior edge of the tibial tray. Black dot: center of the posterior concavity of the tibial tray. **c**. RTC angle is defined as the angle between **C** and **D**. **C**: Line connecting the medial border of the tibial tuberosity with the black dot. **D**: Line perpendicular to the white line.

Twenty-six knees in the version 3.1 group and 25 patients in the version 4.0 group were assessed by axial CT imaging. Rotational alignment of the femoral and tibial components was evaluated by CT. The rotational femoral component angle was defined as the angle between the line through the center of both fixation pegs and the surgical epicondylar axis (Figure 3a). The rotational tibial component angle was defined as the angle between the line connecting the medial border of the tibial tuberosity with the center of the posterior concavity of the tibial tray and the line formed perpendicular to the line along the posterior edge of the tibial tray [18] (Figure 3b, c). The ideal rotational femoral and tibial components angles were defined as falling within 3° of 90° [12].

All radiographs and CT scans were measured twice at 3-month intervals by one observer (HI), who was not part of the operating team and who had no knowledge of the patients.

Operating time was collected from records of intraoperative information. Postoperative KSSs and range of motion were recorded 1 year after surgery.

### Statistical analysis

Data were analyzed using the EXCEL statistics 2008 (SSRI Co., LTD., Tokyo, Japan) software package for Microsoft Windows. Data were checked for normality of distribution using the Kolmogorov–Smirnov test. For normally distributed data Student’s t-test was used to compare the two groups. For data not normally distributed, the Mann–Whitney U-test was applied. Fisher’s exact probability test was used to compare the rate of optimally implanted components between the two groups. All significance tests were two-tailed, and a significance level of P < 0.05 was used for all tests.

## Results

**Figure 4:**
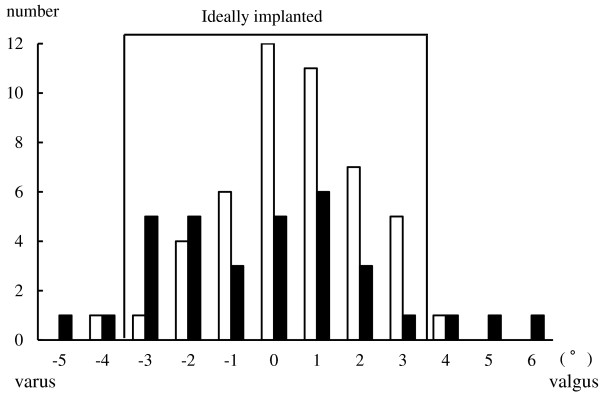
**Distribution of the postoperative hip–knee–ankle angle (=leg mechanical axis).** Box represents ideal alignment ( ± 3° of biomechanical neutral).

The average postoperative HKA angle was 179.8° ± 2.6° [mean ± standard deviation (SD), range: 174°–185°] for the 3.1 group and 179.5° ± 1.5° (range: 176°–184°) for the 4.0 group. With regard to outliers, 28 cases (84.8%) were implanted ideally (within 3° of 180°) in the 3.1 group, whereas 47 cases (96.0%) were implanted ideally in the 4.0 group. There was not a statistically significant improvement in the more advanced navigation system group (P =0.11) (Figure 4). The average FFC angle was 89.8° ± 1.6° (range: 87°–94°) for the 3.1 group and 90.6° ± 1.3° (range: 87°–93°) for the 4.0 group. The average FTC angle was 90.4° ± 1.7° (range: 86°–95°) for the 3.1 group and 90.2° ± 1.3° (range: 87°–93°) for the 4.0 group. The average LFC angle was 5.8° ± 2.7° (range: 0°–12°) for the 3.1 group and 6.7° ± 3.1° (range: 1°–13°) and there was no case of anterior femoral notching in either group. The average LTC angle was 85.5° ± 2.3° (range: 78°–89°) for the 3.1 group and 85.6° ± 1.6° (range: 82°–89°) for the 4.0 group. For the individual component positioning on radiographs, there were no statistically significant findings (Table 2).

**Table 2:** Postoperative component alignment

**(A) Mean degrees of individual component alignment ( ± SD)**			
	**Version 3.1 group**	**Version 4.0 group**	**P- value**
Radiograph			
Number	33	49	
HKA (°)	179.8 ± 2.6	179.5 ± 1.7	n.s
FFC (°)	90.2 ± 1.6	90.6 ± 1.3	n.s
FTC (°)	89.5 ± 1.8	90.2 ± 1.3	n.s
LTC (°)	85.5 ± 2.4	85.6 ± 1.6	n.s
LFC (°)	5.8 ± 2.8	6.7 ± 3.1	n.s
CT			
Number	26	25	
RFC (°)	−0.5 ± 2.3	0.2 ± 2.2	n.s
RTC (°)	−0.2 ± 3.4	1.1 ± 3.4	n.s
**(B) Ideal implantations**			
Radiograph			
HKA	84.8% (28/33)	95.9% (47/49)	n.s
FFC	87.8% (29/33)	91.8% (45/49)	n.s
FTC	87.8% (29/33)	93.9% (46/49)	n.s
LTC	81.8% (27/33)	91.8% (45/49)	n.s
CT			
RFC	80.7% (21/26)	84.0% (21/25)	n.s
RTC	73.1% (19/26)	72.0% (17/25)	n.s

The mean RFC angle was −0.5° ± 2.3° (4° of external rotation to 5° of internal rotation) for the 3.1 group and 0.0° ± 2.1° (4° of external rotation to 5° of internal rotation) for the 4.0 group. Ideal rotational femoral angle was obtained in 80.7% (21 of 26knees) in the 3.1 group and 84.0% (21 of 25 knees) in the 4.0 group. The mean RTC angle was −0.7° ± 3.1° (7° of external rotation to 7° of internal rotation) in the 3.1 group and 0.4° ± 3.3° (7° of external rotation to 5° of internal rotation) in the 4.0 group. Ideal rotational tibial angle was obtained in 73.1% (19 of 26knees) in the 3.1 group and in 72.0% (18 of 25 knees) in the 4.0 group (Table 2). No statistically significant improvement with regard to the rotational alignment was observed. Intra-observer differences were as follows: HKA 0.2° ± 0.4°, FFC 0.2° ± 0.4°, FTC 0.2° ± 0.4°, LFC 0.5° ± 1.0°, LTC 0.4° ± 0.6°, RFC 0.4° ± 0.5° and RTC 0.7° ± 0.7°.

Mean post-operative KSS was 90.1 (range: 72–99) for the 3.1 group and 91.1 (72–100) for the 4.0 group. Mean post-operative functional score was 78.8 (40–100) for the 3.1 group and 77.9 (30–100) for the 4.0 group. No significant difference was detected in these values between the two groups.

Range of motion did not differ between the groups 1 year after surgery.

Operating time was significantly longer in the 3.1 group, with a median duration of 137 minutes (range: 99–209) compared with 125 minutes (range: 90–188) for the 4.0 group (P < 0.01) (Table 3).

**Table 3:** Intra- and post-operative observations

	**3.1 group (N = 33)**	**4.0 group (N = 49)**	**P-value**
Operating time (min)	136.9 ± 22.4	124.9 ± 22.1	0.008
Post-operative (1 year)			
KSS	90.1 ± 6.4	91.1 ± 6.2	n.s
Maximum extension (°)	−1.4 ± 3.2	−2.0 ± 3.4	n.s
Maximum flexion (°)	113.1 ± 11.8	116.6 ± 10.4	n.s

## Discussion

In previous studies, increased rates of early aseptic loosening of TKA were in large part attributed to malalignment of the mechanical axis [4,21]. The tolerable range of mechanical axis deviation after TKA is still under discussion [22]. However, several authors report superior long-term survivorship of TKA with a leg mechanical axis within 3° of the ideal angle [4,21].

In the current study, implants were placed within ± 3° of the desired angle in 96% of the 4.0 group. There have been many publications on computer navigation assisted TKA and its accuracy in terms of the mechanical axis. Our results regarding the accuracy of the mechanical axis using the most recent navigation system (4.0) seemed comparable or superior to those of other studies [11,23]. Molli et al. [16] showed that an earlier navigation system update (from Stryker 2.0 to Stryker 3.1) improved the accuracy of TKA. Several software advancements occurred between Stryker 2.0 and Stryker 3.0 such as a different algorithm for calculating the center of the ankle joint and the addition of “reactive workflow” software. However, our study represented the 4.0 group did not show a statistically significant advantage over the 3.1 group with regard to alignment. This may be quite natural because of the same algorithm for calculation, the same “work flow” software, and the same landmarks to determine the mechanical axis.

With regard to accuracy of the rotational alignment, few studies have demonstrated an improvement with computer-assisted navigation compared with conventional methods [12,13,24]. For femoral rotation, many authors have reported variability in the identification of the transepicondylar axis [25,26]. Yau et al. [26] found that the maximum combined error was 8.2° with 5.3° at the medial femoral epicondyle and 2.9° at the lateral in the transepicondylar axis. Some authors have speculated that this variability is caused by soft tissue coverage [27,28]. However, in a cadaveric study, Siston et al. [29] demonstrated high variability even after all soft tissues had been stripped. Mizu-uchi et al. [12] demonstrated that 89.3% of femoral components were implanted within 3° of ideal rotational alignment in the CT navigation group, whereas 66.7% were implanted ideally in the conventional group. They concluded the CT-based navigation system significantly improved the accuracy of femoral rotational alignment. In the current study 82.3% of femoral components [42 of 51 knees, version 3.1: 80.8% (21/26), version 4.0: 84.0% (21/25)] were implanted ideally. Although no comparison was made with a conventional group in our study, this result is superior to those mentioned in previous studies using conventional techniques [12,15]. Stock et al. [13] noted improvement using a navigation system that established femoral rotational alignment by averaging the angles determined by Whiteside’s line and the transepicondylar axis. The femoral implant was ideally located in over 80% of cases in the current study, possibly because the navigation system used here determines the femoral axis in the same manner. We observed no significant improvement using the system update (from Stryker 3.0 to 4.1).

For tibial rotation, it is much more difficult to define the AP axis, which is the line from the medial border of the tibial tuberosity to the center of the posterior cruciate ligament. In addition, there is no consensus on how to measure rotational alignment of the tibial component. Aiming for the posterior cruciate ligament during surgery is relatively easy, but this structure is difficult to determine on CT scans after implantation. Therefore, we used a line from the medial border of the tibial tuberosity to the center of the posterior concavity of the tibial tray as a reference for rotational alignment. In Mizu-uchi et al. [12], 78.6% of the tibial components were implanted within 3° of the ideal rotational alignment in the CT navigation group, whereas 46.2% of the tibial components were implanted ideally in the conventional group. The CT-based navigation system improved the accuracy of tibial rotational alignment significantly in that study. In this study, 72.5% of the tibial components [37 in 51 knees, version 3.1: 73.1% (19/26), version 4.0: 72.0% (18/25)] were implanted ideally, which is comparable to previous reports [7,12,30]. However, as is often reported in previous studies, the accuracy of the tibial rotational alignment is inferior to that of the frontal, sagittal and femoral rotational alignments [12,30].

The accuracy of the rotational alignment the CT-based navigation reached is ideally enough for us to urge to use it. However, taking the demerits of the additional cost and radiation dose associated with CT into consideration, what we have to do is to improve the accuracy using CT-free navigation system. To achieve more accurate rotational alignment, not only further refinements in navigation technology but also more careful and precise registration will be necessary. Additional reference lines such as a posterior condylar line and a trochlear line may reduce the registration error and improve femoral rotational accuracy [29,31]. Improvements in rotational accuracy may lead to a better postoperative outcome [32].

The navigation system update from Stryker 3.1 to Stryker 4.0 reduced operating time by 12 min. Advancements in navigation software and specific adapted instruments may account for this improvement. One of the disadvantages of using navigation systems in TKA is said to be the increased operating time. In previous studies, time for computer-assisted surgical procedures increased by 8–16 min [16,23,33,34]. Navigation system update may have overcome this problem to some extent.

There were some limitations to the current study. One limitation is that surgery in the two groups was performed at different time periods. Some studies have showed that the learning curve affects operating time and alignment in navigated surgery, especially in early cases. Twenty to thirty implantations were said to be necessary before surgeons were accustomed to the navigation system and the average operating time reached a plateau [35,36]. At our institute, 30 implantations using the image-free navigation system (Stryker 2.0 and 3.1) had been performed by the end of November 2007. Therefore, we think our result would not have been affected by the learning curve. Another limitation is the relatively small number of patients. For instance, with regard to HKA angle, 85% were implanted ideally in the 3.1 group, whereas 96% were implanted ideally in the 4.0 group. Indeed there was not a statistically significant improvement (P =0.11). However, we think there was at least a tendency of improvement and further study may change our conclusion.

## Conclusion

In conclusion, the navigation system update from Stryker 3.1 to Stryker 4.0 reduced operating time by 12 min. However, there were no statistically significant findings with regard to component positioning and clinical outcome.

## Competing interests

The authors declare that they have no competing interests.

## Authors’ contributions

HI designed the study and wrote the draft of the manuscript. ST, KN and HT provided logistical support. ST and TN participated in the design of the study and performed the statistical analysis. ST conceived the study, and participated in its design and coordination. All authors read and approved the final manuscript.

## Pre-publication history

The pre-publication history for this paper can be accessed here:

http://www.biomedcentral.com/2052-1847/5/10/prepub
